# Position paper on undergraduate Palliative Medicine education for doctors in South Africa

**DOI:** 10.4102/phcfm.v14i1.3202

**Published:** 2022-07-07

**Authors:** Henriette Burger, Rene Krause, Charmaine Blanchard, Julia Ambler, Linda Ganca, Alan Barnard, Michelle Meiring, Mpho Ratshikana-Moloko, Hanneke Brits, Tracey Brand, Mitchell Scott, Langalibalele Mabuza, Martin Bac, Nozuko Zele-Mqonci, Parimalaranie Yogeswaran, Liz Gwyther

**Affiliations:** 1 Division of Radiation Oncology, Faculty of Medicine and Health Sciences, Stellenbosch University, Cape Town, South Africa; 2 Division of Radiation Oncology, Tygerberg Academic Hospital, Cape Town, South Africa; 3 Division of Family Medicine, Faculty of Health Sciences, University of Cape Town, Cape Town, South Africa; 4 Centre for Palliative Care, Faculty of Health Sciences, University of the Witwatersrand, Johannesburg, South Africa; 5 Non-Communicable Diseases Research Unit, WITS Health Consortium, Johannesburg, South Africa; 6 Department of Paediatrics, School of Clinical Medicine, University of KwaZulu-Natal, Durban, South Africa; 7 Umduduzi Hospice Care for Children, Pietermaritzburg, South Africa; 8 Department of Paediatrics and Child Health, Faculty of Health Sciences, University of Cape Town, Cape Town, South Africa; 9 Paedspal, Cape Town, South Africa; 10 Non-Communicable Diseases Research Unit, WITS Health Consortium, Johannesburg, South Africa; 11 Division of Family Medicine, Faculty of Health Sciences, University of the Free State, Bloemfontein, South Africa; 12 Department of Family Medicine, School of Clinical Medicine, University of KwaZulu-Natal, Durban, South Africa; 13 Department of Family Medicine and Primary Health Care, Faculty of Health Sciences, Sefako Makgatho Health Sciences University, Ga-Rankuwa, South Africa; 14 Department of Family Medicine, Faculty of Health Sciences, University of Pretoria, Pretoria, South Africa; 15 Department of Family Medicine, Faculty of Health Sciences, Steve Biko Academic Hospital, Tshwane, South Africa; 16 Department of Family Medicine and Rural Health, Faculty of Health Sciences, Walter Sisulu University, Mthatha, South Africa

**Keywords:** Palliative Medicine, palliative care, curriculum design, education, health professions education, learning outcome, competencies

## Abstract

**Background:**

Basic palliative care teaching should be included in training curricula for health care providers (HCPs) at all levels of the health service to ensure that the goal set by the South African (SA) National Policy Framework and Strategy for Palliative Care, to have an adequate number of appropriately trained HCPs in South Africa, is achieved. Furthermore, palliative learning objectives for nurses and doctors should be standardised. Many SA medical schools have integrated elements of Palliative Medicine (PM) teaching into undergraduate medical training programmes for doctors; however, the degree of integration varies widely, and consensus and standardisation of the content, structure and delivery of such PM training programmes are not yet a reality.

**Aim:**

This joint position paper aims to describe the current state of undergraduate medical PM teaching in South Africa and define the PM competencies required for an SA generalist doctor.

**Setting:**

Palliative Medicine programme leads and teachers from eight medical schools in South Africa.

**Methods:**

A survey exploring the structure, organisation and content of the respective medical undergraduate PM programmes was distributed to PM programme leads and teachers.

**Results:**

Responses were received from seven medical schools. Through a process of iterative review, competencies were defined and further grouped according to suitability for the pre-clinical and clinical components of the curriculum.

**Conclusion:**

Through mapping out these competencies in a spiralled medical curriculum, the authors hope to provide guidance to medical curriculum designers to effectively integrate PM teaching and learning into current curricula in line with the goals of the SA National Policy Framework and Strategy on Palliative Care (NPFSPC).

## Introduction

**FIGURE 1:**
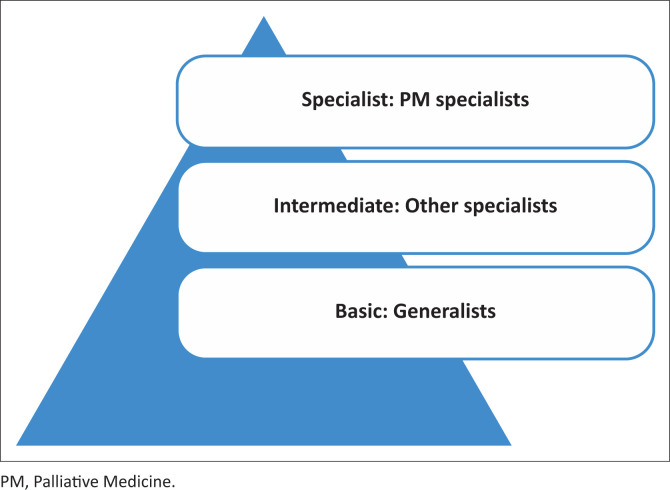
Levels of palliative care training for doctors according to the scope of practice.

The 2014 World Health Assembly (WHA) Resolution 67.19, ‘Strengthening of palliative care (PC) as a component of comprehensive care throughout the life course’, describes PC as an approach that improves the quality of life of patients (adults and children) and their families, who face life-threatening illness, by preventing and alleviating unnecessary suffering.^1^ The Resolution advocates for the integration of PC services across all levels of the health system and continuum of care, with a specific focus on primary care. It urges member states to include PC as an integral component of the basic and ongoing education and training offered to medical and nursing professionals and outlines three levels of PC training according to their scope of practice (Figure 1).^1^

In response to the Resolution, the South African (SA) National Department of Health approved its National Policy Framework and Strategy on Palliative Care in 2017.^2^ One of the five overarching goals of the policy is to ensure adequate numbers of appropriately trained healthcare providers (HCPs) to deliver PC at all levels of the health service. An educational objective defined for this goal is ensuring that basic Palliative Medicine (PM) and PC teaching is included in the undergraduate/pre-service and post-graduate curricula of all HCPs. This includes standardising palliative learning objectives for nurses and doctors.^2^ For the purposes of this article, the term Palliative Medicine (PM) refers to the clinical care provided by doctors trained in the discipline, whilst PC is the broader approach provided by doctors, nurses, psychosocial or spiritual counsellors and other members of an interdisciplinary team.

Most universities in South Africa have responded to this fundamental training need by integrating basic PM teaching into their undergraduate medical curricula drawing on international and regional consensus documents intended to guide institutions in delivering standardised evidence-based PM teaching programmes for medical students.^3,4,5,6,7^ The level of integration, however, varies greatly and consensus, and standardisation of the content, structure and delivery of such undergraduate medical PM training programmes are not yet a reality.^8^

This position paper represents a joint endeavour by the PM leads from the eight medical schools in South Africa – University of Cape Town (UCT), University of the Free State (UFS), University of KwaZulu-Natal (UKZN), University of Pretoria (UP), Stellenbosch University (SU), University of the Witwatersrand (WITS), Walter Sisulu University (WSU) and Sefako Makgatho Health Sciences University (SMU) – to meet this objective for undergraduate medical education. The objectives of the collaboration were to (1) describe the current state of undergraduate medical PM teaching in South Africa, (2) define the PM competencies required of an SA generalist doctor and (3) map out these competencies in a spiralled medical undergraduate curriculum.

## Current state

To ascertain the state of PM training at the eight SA medical schools, the collaborating authors completed a survey exploring the structure, organisation and content of their respective undergraduate palliative medicine programmes (UPMP) in May 2019. Responses were received from seven universities (UCT, UFS, UKZN, UP, SU, WITS and SMU). The curriculum specifications of the seven adult and two paediatric PM programmes (UCT and UKZN) are summarised in Table 1.

**TABLE 1:** Curriculum specifications.

Curriculum specification†	*n*	%	Median	Range
**Dedicated UPMP**	7	77.8	-	-
**Integration of UPMP**			-	-
Stand-alone	2	22.2	-	-
Integrated	7	77.8	-	-
**Hours dedicated to UPMP (Adult and paediatrics combined)**	-	-	27.5	6–46
**UPMP integrated in pre-clinical and clinical years**	4	44.4	-	-
**Dedicated PM clinical contact time**	3	33.3	-	-
**Highest PM qualification held by faculty**				
PhD	1	11.1	-	-
Master’s in PM	3	33.3	-	-
PG Diploma in PM	4	44.4	-	-
None	1	11.1	-	-
**External lecturers contracted**	4	44.4	-	-
**Interdisciplinary faculty in addition to doctors**				
Social worker	6	66.7	-	-
Registered nurse	2	22.2	-	-
Other (spiritual counsellors)	2	22.2	-	-
**Content domains represented in programmes**				
Pain management	9	100.0	-	-
Psychosocial care	9	100.0	-	-
Basic Principles and Practice of PC	8	88.9	-	-
Symptom management	8	88.9	-	-
Communication	8	88.9	-	-
Terminal/end-of-life care	8	88.9	-	-
Ethical and legal aspects of PM	8	88.9	-	-
Spiritual care	7	77.8	-	-
Grief and bereavement care	6	66.7	-	-
Cultural sensitivity	6	66.7	-	-
Teamwork in PM	6	66.7	-	-
Self-reflection and self-care	4	44.4	-	-
**Assessment methods**				
Formative	4	44.4	-	-
Summative	8	88.9	-	-

*N* = 9.

PM, Palliative Medicine; PC, pallaitive care; PhD, Doctor of Philosophy; PG, postgraduate; UPMP, undergraduate Palliative Medicine programmes.

† , Adult and paediatrics programmes separated unless specified. Does not include data from Walter Sisulu University.

Seven respondents stated that a dedicated UPMP was already established at their institution. Programmes were led by doctors, all of whom had postgraduate qualifications in PM, except for SMU. In two programmes, a member of the team held a postgraduate qualification in Health Professions Education. Across programmes a median of three team members had postgraduate (PG) PM qualifications (range 1–7). Interdisciplinary faculty was mostly represented by social workers with only two programmes using nurses and spiritual counsellors to train students. The services of external PC-trained lecturers for teaching or assessment were necessitated at four institutions.

Palliative Medicine teaching was most often integrated into other modules, including Family Medicine, Internal Medicine, Paediatrics, Public Health and pre-clinical modules on professional clinical practice. When combining adult and paediatric UPMP teaching hours across the degree course (median: 27.5 h, range: 6 h – 46 h), only one institution, UCT, met the 40 h recommended by the European Association of Palliative Care to achieve basic undergraduate PC competency.^5^ Palliative medicine was taught in both the pre-clinical and clinical phases of undergraduate education in only four programmes. Undergraduate Palliative Medicine programmes employed a wide range of teaching and learning activities, with formal lectures and clinical bedside teaching being the most common. Only three UPMP included dedicated clinical contact time like rotation through a hospice or PC ward.

The content domains of pain management and psychosocial care were included in all programmes. Additional content domains included in at least seven of nine programmes were basic principles and practice of PC, symptom management, end-of-life care, ethical and legal issues, communication skills and spiritual care. Formative assessment was used in four programmes, and summative assessment in all but one. Student feedback on the UPMP, predominantly in the form of anonymous questionnaires, was used as a means of quality improvement at seven institutions.

A few programmes needed to augment their funding through support from non-governmental organisations (NGOs) and provincial departments of health. All but one respondent stated that their UPMP had established teaching partnerships with local hospices, private practices or PC organisations.

## Defining palliative medicine competencies

In a national survey to define essential PC competencies for medical students in the United States of America, Shaeffer et al. asked PC educational experts to rate the importance of 18 competencies that were adapted for graduating medical students from recognised postgraduate PM training programmes.^9^ The majority of respondents (50.1% – 67.6%) rated the following seven competencies as essential for all graduating medical students: ethical decision-making, personal reactions to death and dying, identifying psychosocial distress, exploring patients’ disease understanding and goals for care, the role of PC across the life cycle and patient-centred communication.^9^ As a first step towards defining the required PM competencies of the SA medical graduate, the collaborating PM programme leaders were asked to rate the importance of the same list of 18 competencies. Table 2 shows the rating results of those competencies considered very important by more than five of the nine programme leads. It indicates the relative emphasis SA PM leads place on psychosocial and spiritual care as well as symptom management skills. Secondly, the collaborators were asked to review the collated survey results and formulate PM competencies relevant to the SA setting. Through a process of iterative review, competencies were defined and further grouped according to suitability for the pre-clinical and clinical setting. Table 3 presents these competencies in a spiralled structure that serves to progressively build knowledge and confidence in the practical application of PC skills. Notably none of the programme leads selected ‘not important’ as a response for any of the competencies reviewed.

**TABLE 2:** Rating of selected† palliative care competencies.

Domain	Palliative care competencies for graduating medical students	Very important	Somewhat important
PC principles	Reflects on personal emotional reactions to patients’ dying and deaths	9	0
	Describes the roles of members of an interdisciplinary PC team	9	0
Psychosocial care	Identifies psychosocial distress in patients and families	9	0
	Identifies patients’ and families’ cultural values, beliefs and practices related to serious illness and EOL care	9	0
	Identifies spiritual and existential suffering in patients and families	9	0
	Describes an approach to the diagnosis of anxiety, depression and delirium	7	2
Symptom management	Assesses pain systematically and distinguishes nociceptive from neuropathic pain syndromes	9	0
	Assesses non-pain symptoms and outlines a differential diagnosis, initial workup and treatment plan	9	0
	Identifies common signs of the dying process and describes treatments for common symptoms at the EOL	9	0
	Describes key issues and principles of pain management with opioids, including equianalgesic dosing, common side effects, addiction, tolerance and dependence	7	2
Communication	Demonstrates patient-centred communication techniques when giving bad news and discussing CPR preferences	9	0
	Explores patient and family understanding of illness, concerns, goals and values that inform the plan of care	8	1
	Demonstrates basic approaches to handling emotion in patients and families facing serious illness	7	2
Ethics	Describes ethical principles that inform decision-making in serious illness	6	3

*Source:* Schaefer KC, Chittenden, EH, Sullivan AM, et al. Raising the bar for the care of seriously ill patients: Results of a National Survey to define essential palliative care competencies for medical students and residents. Acad Med. 2014;89(7):1024–1031. https://doi.org/10.1097/ACM.0000000000000271

† , Very important by > 5 respondents.

PC, palliative care; EOL, end-of-life; CPR, cardiopulmonary resuscitation.

**TABLE 3:** Spiral structure of palliative care competencies.

Content domain	Pre-clinical phase: Competencies	Clinical phase	
		Competencies	Suggested learning method in clinical phase
Principles of palliative care	To understand the principles of PC as summarised in the WHO definition including the importance of spiritual support	To apply the principles of PC in care planning	Develop a comprehensive palliative care plan from a thorough PC assessment
Communication skills	To be able to break bad news sensitively, respond to emotion and build realistic hope	To be able to discuss goals of care in serious illness, including advance care planning, demonstrating sensitivity to the individual, recognising culture and personal preferences of the patient and family (year 6)	Basic application in an observed simulated environment. Feedback from peers and educators is advised.
Interdisciplinary teamwork	To understand the structure and roles within an interdisciplinary team (IDT)	To demonstrate the ability to function as part of an IDT	Participate in IDT meetings Include IDT in care planning
Bioethical principles ethics	To understand bioethical principles as applied to palliative care	To be able to identify ethical dilemmas (year 4) and apply an ethical approach to manage identified dilemmas, for example, withholding or withdrawing treatment (year 6)	Apply in care planning
Integration of PC in the health system	Knowledge of current PC service platforms	To understand how PC service provision is integrated at different levels of the health system	Demonstrate the ability to write a comprehensive referral to a PC service
End-of-life care	To understand the normal dying process and the psychosocial effect it has on the patient and family	To be able to identify the dying phase (year 4) and manage this appropriately with attention to all aspects of care (year 6)	Develop an end-of-life care plan
Grief and bereavement	To be able to recognise normal grief and provide basic bereavement support for adults and children (year 3)	To be able to identify complicated (year 4) grief and refer to appropriate support services (year 6)	Assessment and basic inclusion in care planning
Support of the family	To understand the impact of serious illness on family members	To be able to provide support to family members of PC patients of all ages, including having an approach to conducting a family meeting (year 6)	Participate in family meetings. To include the family in care planning
Focus areas in paediatric PM	To describe the impact of a serious or chronic illness on the child, their family, the school, the community, the health system and the health care practitioner To apply Piaget’s stages of cognitive development to the child’s understanding of illness at different stages	To understand the impact of serious illness within the paediatric context To understand the assessment and management of the most common symptoms in children To understand how the child’s understanding of illness, death and dying develops	Develop a palliative care plan for a child with a serious illness
Self-care and positive resilience	To understand the concepts of cumulative grief, compassion fatigue, countertransference and burnout and positive resilience	To demonstrate self-reflective practice and the ability to build positive resilience	Develop self-care plan identifying own strengths, weaknesses, triggers and warning signs and listing individualised activities aimed at building resiliency
Cultural awareness	To recognise variance in the cultural understanding of PC and how to approach PC provision in a culturally appropriate and acceptable manner	To incorporate respect for cultural differences in palliative and end-of-life care and communication	Apply in cross-cultural care planning and communication activities
Concept of total pain (biopsychosocial and spiritual)	To understand the concept of total pain as it defines HRS and to recognise the aspects of HRS in South Africa	To be able to do a palliative care assessment, which includes a biopsychosocial and spiritual needs assessment of the patient and family (if the patient is unable to give a history, obtain from the caregiver) (year 4) To be able to develop an appropriate care plan for each individual patient and to extend this to discharge planning, ensuring continuity of care for the palliative care patient (year 6) To be able to provide basic counselling support for psychosocial and spiritual problems and to refer to appropriate practitioner	Develop a comprehensive palliative care plan from a thorough PC assessment
Identification of patients with PC needs		To be able to recognise patients who would benefit from palliative care across the disease spectrum (adults and children) using validated tools	Demonstrate the ability to write a comprehensive referral to a PC service
Pain and symptom control	Anatomy and physiology of pain conduction	To be able to assess pain (year 4) and manage pain using basic pharmacological and non-pharmacological interventions and management of pain that is difficult to control including referral to appropriate clinician/service	Simulate a prescription for strong opioids.
		To be able to assess (year 4) and manage (year 6) other distressing symptoms with pharmacological and non-pharmacological modalities, including: ***Essential:*** dyspnoea, delirium, nausea and vomiting, constipation, cachexia, anorexia and fatigue, anxiety ***Important:*** diarrhoea, mouth care, pressure sores, cough, hiccups lymphoedema, depression, wound care	Include pharmacological and non-pharmacological management in the care plan
PC emergencies		To be able to identify (year 4) and manage (year 6) palliative care emergencies	Case report on Palliative emergency observed during acute care rotations in emergency unit

Note: Where relevant, appropriate year numbers are indicated in brackets.

WHO, World Health Organisation; PC, palliative care; HRS, health-related suffering.

## Locally relevant content

The PM curriculum should respond to the specific needs of the SA population and address the unique morbidity and mortality profile resulting from its quadruple disease burden of maternal and child mortality, communicable diseases, non-communicable diseases and trauma. South African medical graduates need to be able to make ethical decisions considering limited resources, culturally diverse needs and the right of every citizen to good PC. Communication skills training should take our multilingual society and cultural and spiritual diversity into account. Care planning for patients and families from disparate socioeconomic backgrounds must be individualised according to the level of support required and available resources. Graduates need to understand the value that each member of the interdisciplinary team adds to patient care and be flexible and innovative when managing limited resources to the benefit of their patients.

## Golden threads

Most medical curricula follow a spiralling structure of learning, where knowledge, skills and attitudes progressively build year on year both in the depth of understanding and in the level of application. The authors recommend that the following golden threads be spiralled throughout the entire medical curriculum in an integrated way:

- Applying the principles of PC^10^ and patient centredness in care across the life course, from birth to death.
- Understanding the concept of total pain as it defines health-related suffering in the physical, social and spiritual domains of living.
- Treating severe pain using pharmacological and non-pharmacological methods, including the ability to prescribe opioids confidently and safely for severe pain in all age groups.
- Communication skills to facilitate engagement with patients, family members and colleagues in the health care team.
- Cultural awareness and sensitivity in all aspects of care provision.
- Implementation of a team approach in PM.
- Prioritising self-care and self-reflective practice, which should include an understanding of their own attitude towards death.

## Integration in the pre-clinical years

Our survey revealed that most PM teaching hours are positioned in the clinical years (4–6) and tend to be integrated into established rotations dedicated to disciplines like family medicine, internal medicine and paediatrics. Teaching and learning activities are mostly case based, and therefore comprehensive coverage of basic PM theory cannot be guaranteed. Current activities include patient narratives, paper cases and reflection on own experiences. The apparent lack of focus on theoretical PM teaching in the pre-clinical years is of concern. Feedback received by the PM leads from undergraduate students indicates feelings of helplessness and moral distress experienced by the students when confronted with dying patients with uncontrolled symptoms or who are not cared for with dignity. This exposure to patients who are perceived to be suffering greatly can negatively impact young clinicians who have not been adequately prepared for such situations and lack the medical skills and authority to intervene. The authors believe that the theoretical elements and skills of PM should be integrated in the pre-clinical years to equip students for patient interactions in their subsequent clinical exposure. This includes a thorough grounding in the principles of Palliative Medicine including the concept that in most cases, dying can be regarded as a normal process and that relief from pain and other distressing symptoms can be provided without hastening or postponing such a death.

In most undergraduate medical curricula in SA, the first 2–3 years consist predominantly of theoretical learning with only limited exposure to patients and the clinical environment. Teaching is focused on basic medical sciences including anatomy, physiology, microbiology and pathology. More recently, this has extended to broader domains including health systems, health promotion and disease prevention, professionalism, clinical reasoning and decision-making, scholarship and research methods, health advocacy, teamwork, self-care and clinical communication. These domains aim to develop engaged practitioners with a holistic approach to care who understand their role in the health system and in the communities they serve.

Considering that the pre-clinical years are already quite content heavy, the authors recommend a fully integrated approach where the pre-clinical competencies listed in Table 3 are built into existing modules. Teaching on health systems should acknowledge that many diseases, especially in the SA context, cannot be cured and that universal health coverage should ensure holistic support and symptom relief for patients with serious illnesses and their families. Students should be challenged to explore where PM can be integrated into the different levels of local health service provision, including the role of NGOs and volunteers in the community in accordance with the WHA resolution. Physiology modules can easily extend their reach to include pain physiology and physiology of other end-of-life symptoms, the dying process and normal grief. Communication skills training should introduce students to conversation tools like the SPIKES (setting, perception, invitation, knowledge, empathy, summary) protocol that can guide them to break bad news compassionately and respond to the emotions expressed by patients and their families with professional empathy.^11^ Students should also learn techniques to explore patients’ care preferences and goals through learning activities like role-playing. Many modules on professionalism already address interdisciplinary teamwork and self-awareness. Here the structure and functioning of an interdisciplinary PC team could be studied as a practical example to emphasise the importance of a team approach in PM. The psychological repercussions for the health care worker after the death of a patient need to be acknowledged before the advent of the clinical years, especially in a setting such as South Africa with high rates of maternal and child mortality and preventable deaths from trauma, violence and suboptimal health care delivery.^12,13^ This can be sensitively integrated into modules on self-awareness and self-care. Teaching on cultural sensitivity and multilingualism has become essential in SA undergraduate medical curricula. Again, this theme can be easily extended to small group discussions where students can explore the differences in their cultural understanding of illness, death and dying and funeral rituals amongst peers.

Such an integrative approach to curriculum design would require a detailed overview of the entire curriculum to avoid unconsidered duplication or contradictions in content and to ensure the progressive building of knowledge both within the pre-clinical years and beyond.

## Integration in the clinical years

The clinical years transition the medical student from the classroom into the hospital, clinic and patient’s home where clinical competencies can be developed through direct personal interaction with patients. Here the focus should shift to the practical application of PC principles and tools in all clinical disciplines. Cross-cutting activities such as screening patients for PC needs, pharmacological and non-pharmacological symptom management, ethical decision-making, intentionally engaging with families, discharge planning, ensuring continuity of care across platforms and functioning as part of interdisciplinary teams should be practised in a variety of clinical settings. Bedsides training in hospitals, hospices, intermediate care facilities and patients’ homes provide opportunities for students to apply the theory of PM in a patient encounter. Students would be encouraged to reflect on the complexities of working in different settings and develop an understanding of the continuity of care. Palliative medicine teaching and learning are mainly situated within Family Medicine programmes in the current SA setting. However, many universities integrate the palliative approach into paediatrics, internal medicine and the surgical rotations.

Clinical PM competencies are based on completing a comprehensive PC assessment followed by developing a care plan for individual patients that are aligned with their goals and preferences for care. The assessment should include the assessment of biopsychosocial and spiritual needs. Good communication skills, understanding of progressive disease and PC symptoms and the ability to develop a clinical, psychosocial and spiritual care plan are needed.

Small group problem-based learning can be used effectively to teach an approach to symptom management (see Table 3) in progressive incurable diseases.^14^ This is a skill that students should master before graduation. Students can practise the integration of clinical assessment and symptom management by drawing up a care plan for a patient with advanced organ failure or cancer. The clinical care plan can be assessed by evaluating the degree to which the student is able to correctly identify and manage the patient’s symptom using a pharmacological and non-pharmacological approach. Golden threads such as team involvement, ethical decision-making and PC principles should be woven through the care plans, thereby building on the pre-clinical learning proposed above.

Working with vulnerable adults, children and their families in PC can be technically and emotionally challenging for students. Supervised clinical sessions and small group tutorials are teaching methods that are recommended to normalise the PC experience and provide participants with the personal skills to become resilient. Reflective teaching in small groups may help students develop not only their technical knowledge and skills but also a compassionate attitude and an ethical compass when managing patients who are facing a life-threatening illness. These sessions are vital to containing students emotionally, and probing questions are used to deepen their reflections and to allow for critical thinking around PC practices.^14^ Additional competencies in paediatric PC should include communicating with children of different ages and developmental stages, assessing and managing pain and other common symptoms in children and supporting parents and siblings.^15^

The assessment of learning of clinical PC skills should form part of summative assessments. Methods of assessment can include patient and family care plans and objective structured practical examination (OSPE) sessions where communication skills, management of palliative emergencies and symptom management can be assessed. Summative assessment may also include written exams with short answer questions and multiple-choice questions.

## Conclusion

As PM is a cross-cutting discipline applicable to all health care settings, it is imperative that doctors within all levels and disciplines of the health service are equipped to provide basic PC services to patients and families affected by a serious or life-threatening illness in their care. This includes having the knowledge and skills to identify patients who need PC and to assist them and their families ‘to live as actively as possible’^10^ and to enjoy good quality of life despite their illness.

A vital step towards ensuring this is the development of a framework of standardised objectives and competencies to guide universities in developing PM curricula for medical students which should be integrated into the various specialties with a particular focus on primary and public health care. This position paper has been compiled by experienced PM teachers and provides guidance to health sciences or medical faculties to effectively integrate PM teaching and learning into current medical curricula in line with the goals of the SA National Policy Framework and Strategy on Palliative Care. We are optimistic that this article will stimulate a similar assessment of PC curricula for other health care disciplines.

This article describes the current state of undergraduate PM teaching for doctors and provides a consensus statement from the eight SA medical schools on basic PM competencies required for the curriculum. Recommendations are provided regarding learning domains, objectives and locally relevant PM content to be included in curricula. The article also suggests methodologies for the integration of PM content into the learning spiral of existing undergraduate medical programmes. This will ensure that graduates have the competencies to care for patients and their families facing a life-threatening illness and to develop their own positive resilience as professionals providing PC in the SA healthcare system.
